# Early Complications of Extracorporeal Shockwave Lithotripsy in The Records of The Department of Paediatrics, Nephrology and Allergology of The Military Institute of Medicine – Preliminary Results

**DOI:** 10.34763/devperiodmed.20182203.260264

**Published:** 2018-10-04

**Authors:** Katarzyna Dobrowiecka, Jędrzej Przekora, Katarzyna Jobs, Katarzyna Kowalczyk, Katarzyna Plewka, Aleksandra Paturej, Bolesław Kalicki

**Affiliations:** 1 Student Scientific Society of the Department of Paediatrics, Nephrology and Allergology of the Military Institute of Medicine, Warsaw, Poland; 2 Department of Paediatrics, Nephrology and Allergology of the Military Institute of Medicine, Warsaw, Poland

**Keywords:** Urolithiasis, children, early complications, extracorporeal shock wave lithotripsy, ESWL, kamica nerkowa, dzieci, wczesne powikłania, litotrypsja zewnątrzustrojową falą uderzeniową, ESWL

## Abstract

The aim of the study was to analyse the frequency and type of early complications of extracorporeal shock wave lithotripsy (ESWL) and to assess the safety of the procedure among paediatric patients. The study is a retrospective analysis of all ESWL procedures carried out due to urolithiasis in 2009-2015 in the Department of Paediatrics, Nephrology and Allergology of the Military Institute of Medicine. Medical records of 170 children were analysed: 94 girls (55.3%) and 76 boys (44.7%) aged 1 to 18 years. Out of the 272 procedures performed, 247 were included in the study. Among 233 effective ESWL treatments, complications were noted in 35 (15.02%) procedures: among 10 girls (40%) and 15 boys (60%). In 25 cases (10.73%) surgical intervention was necessary due to urinary obstruction caused by a fragment of a disintegrated stone. Urinary tract infection was present among 3 patients (1.29%) who required surgical treatment. Nonsurgical complications included 7 urinary tract infections (3%) and 3 cases of severe abdominal pain (1.29%). Complications such as perirenal haematoma, subcapsular haematoma or ureteral perforation were not observed. The vast majority of complications observed in the study were associated with urinary obstruction caused by partial blockage of the ureter with kidney stone fragments (“steinstrasse”). Despite the complications that were documented, ESWL should be considered a safe procedure.

## Streszczenie

Celem pracy jest analiza rodzaju i częstości wczesnych powikłań po zabiegu litotrypsji zewnątrzustrojową falą uderzeniową (ESWL) oraz ocena bezpieczeństwa zabiegu u pacjentów pediatrycznych. Badanie jest retrospektywną oceną dokumentacji medycznej dzieci hospitalizowanych w Klinice Pediatrii, Nefrologii i Alergologii Dziecięcej Wojskowego Instytutu Medycznego w latach 2009-2015 zakwalifikowanych do zabiegu litotrypsji z powodu kamicy układu moczowego. Oceniono dane 170 dzieci, w tym 94 (55,3%) dziewczynek i 76 (44,7 %) chłopców w wieku od 1. do 18. roku życia. Spośród 272 zabiegów, do analizy włączono 247 wykonanych zabiegów litotrypsji. Wśród 233 wykonanych skutecznie zabiegów litotrypsji zewnątrzustrojową falą uderzeniową odnotowano 35 powikłań (15,02%), które wystąpiły u 10 (40%) dziewczynek i 15 (60%) chłopców. W 25 przypadkach (10,73%) pacjenci wymagali interwencji chirurgicznej z powodu zablokowania dróg moczowych fragmentem rozbitego złogu. U 3 dzieci (1,29%) wymagających interwencji chirurgicznej stwierdzono równocześnie infekcję układu moczowego. Pozostałe powikłania nie wymagały interwencji chirurgicznej, było to: 7 zakażeń układu moczowego (3%) oraz 3 przypadki silnych objawów bólowych (1,29%). Nie obserwowano innych wczesnych powikłań takich jak: krwiaki okołonerkowe, krwiaki podtorebkowe czy perforacja moczowodu. Większość obserwowanych w badanym materiale wczesnych powikłań była związana z utrudnieniem odpływu moczu spowodowanym częściową blokadą moczowodu przez pokruszone fragmenty kamienia („steinstrasse”). Pomimo opisanych powikłań zabieg ESWL w badanym materiale należy uznać za bezpieczny.

## Introduction

Urolithiasis is a condition caused by the accumulation of deposits in the urinary tract which form when the concentration of soluble substances in urine exceeds the solubility threshold.

An increase in the incidence of urolithiasis was observed in recent years among both the adult and paediatric population, especially among newborns and infants. The prevalence of the disease ranges from 5% to 9% among adults in European registers [1]. Children represent approximately 2-3% of the total population of the affected patients [2]. However, this number is most likely underestimated, as some studies suggest that up to 10% of the patients diagnosed with urolithiasis are children [3].

Children rarely present with classic symptoms of renal colic: sudden, acute, permanent or gripping pain located in the lumbar or hypogastric region, radiating to the groin, nausea and vomiting. Among the paediatric population symptoms are usually nonspecific and dependent on the patient’s age. The diagnosis of urolithiasis should be considered when a child presents with symptoms such as anxiety, abdominal pain, vomiting, fever, haematuria, recurrent urinary tract infections, spontaneous expulsion of kidney stones [4]. Usually urolithiasis does not lead to chronic kidney disease. However, bilateral or unilateral (in the case of a solitary kidney) urinary obstruction may lead to an irreversible loss of renal function.

Treatment of urolithiasis focuses on evacuation of the deposits and prevention of recurrence. Equally important is the protection of kidney function, prevention of urinary tract infections and correction of urinary tract anatomical aberrations. Furthermore, existing metabolic disorders must be diagnosed in order to ensure adequate treatment [3].

Conservative treatment of renal colic focuses on pain relief, appropriate fluid therapy and treatment of urinary tract infections. Painkillers and antispasmodic drugs are used. Most kidney stones under 4 mm pass spontaneously [5].

Prophylaxis includes adequate fluid supply, appropriate diet, regular physical activity and pharmacotherapy that accounts for detected metabolic disorders. If necessary, crystallization inhibitors should be administered [1, 3].

Invasive procedures should be considered once conservative treatment is insufficient, usually when kidney stones exceed 5 mm in diameter. Minimally invasive procedures include extracorporeal shock wave lithotripsy (ESWL), ureteroscopic lithotripsy (URSL), percutaneous nephrolithotomy (PCNL) and retrograde intrarenal surgery (RIRS) [6]. These methods have almost entirely replaced the classic surgical procedures. The patients’ condition and diagnostic imaging should be taken into consideration upon deciding the method of treatment.

ESWL remains the method of choice for treating kidney stones measuring 6 to 20 mm and ureteral stones smaller than 10 mm. Exceptions include uric acid stones, for which pharmacological treatment is a method of choice. Absolute contraindications to ESWL include abdominal aortic aneurysm, renal artery aneurysm, pregnancy, ongoing urinary tract infection, haemorrhagic diathesis, anticoagulant therapy, bone deformities that prevent the patient from settling onto the exam table, urinary obstruction distant to the deposit (stenosis of the ureteropelvic junction, renal calyx, urethra and benign prostatic hyperplasia [7]).

ESWL uses high energy shock waves generated outside the patient’s body to disintegrate the deposit into smaller fragments. The procedure is usually effective if urine flow is unrestrained and deposits are located in the renal pelvis, upper and middle renal calyx, ureteropelvic junction and the upper part of the ureter [6]. Calculi located in the lower pole of the kidney are frequently difficult to excrete.

Complications of ESWL are a result of kidney stone displacement and fragmentation and of the direct impact of shock waves on the tissues. Early complications include skin lesions (reddening, bruising, petechia, necrosis), oedema of the renal parenchyma, haematomas, haematuria, proteinuria, dilation of the calyceal-pelvic complex due to urinary retention and hydronephrosis, “steinstrasse”, urinary tract infection. These complications are mostly temporary [6]. Actually, minimally invasive therapeutic options are used to treat most complications; it is mainly ureterorenoscopic lithotripsy (URSL) that is performed.

ESWL does not always lead to complete stone clearance. Residual fragments can be the cause of the recurrence of the disease. Late complications of ESWL such as renal hypoperfusion and renal hypertension have not been studied thoroughly, yet there is no evidence that they occur in the paediatric population. [8].

The aim of the study was to retrospectively analyse the frequency and type of early complications and to assess the safety of the ESWL among patients treated in the Paediatrics, Nephrology and Allergology Department of the Military Medical Institute in the years 2009-2015.

## Material and methods

Medical records of 272 ESWL procedures carried out in 2009-2015 in the Department of Paediatrics, Nephrology and Allergology of the Military Institute of Medicine were analysed retrospectively. Data from 25 procedures were disqualified, due to ineffective treatment – no disintegration of kidney stones was obtained, and only the remaining 247 procedures were considered.

The study group consisted of 170 children: 94 girls (55.3%) and 76 boys (44.7%) aged 1 to 18 years (mean 10 years). Body weight ranged from 9.9 kg to 126.9 kg (mean 39.3 kg).

All the procedures were performed with the LITHOSKOP - Siemens multi-function lithotripter. The number of shock waves and their energy were individually adjusted to the age and weight of the patients treated. The range of energy used was 10-19 kV, the number of pulses 15003000 and wave frequency 70-90/min. All the treatments were performed under general anaesthesia.

The post-procedural protocol included hydration, initially intravenous, followed by oral. Fluids were administered according to the patient’s daily requirements, diuresis was not forced and no diuretics were used. In the first days following the procedure, intravenous antispasmodic drugs (drotaverine) and oral alpha-blockers were administered to facilitate the expulsion of defragmented kidney stones. Antibacterial drugs were also used; their type was selected taking into consideration the clinical image.

The procedure was considered effective if the patient expelled fragmented kidney stones within 3 days after the ESWL. As our focus was on early postprocedural complications, the stone-free rate was not determined (the success rate should be evaluated at least 3 months after ESWL).

## Results

Out of the 247 ESWL treatments analysed, 14 were treated as ineffective, as the patients did not expel kidney stone fragments despite their disintegration. The effectiveness in the study group was 94.3%. Only the 233 cases of effective treatments were further analysed.

A total of 35 complications (15.02%) occurred among the effective treatments, they affected 10 girls (40%) and 15 boys (60%). Among 25 patients (10.73%) endoscopic surgical intervention (URSL) was necessary due to urinary tract obstruction caused by a fragment of a disintegrated stone. Two patients were additionally treated with double-J stent placement.

Nonsurgical complications included 7 cases of urinary tract infections (3%), 3 cases of severe abdominal pain (1.29%). Urinary tract infection was also present among 3 patients (1.29%) who required surgical treatment.

No significant early complications such as: perirenal hematoma, subcapsular haematoma or ureteral perforation were observed within the analysed group. As for mild haematuria, it was present in all the cases treated.

## Discussion

Extracorporeal shock wave lithotripsy is currently considered to be the method of choice in the treatment of appropriate uncomplicated cases of urolithiasis in children. It is considered to be safe and effective, however, energy levels and the number of shock waves have to be watched carefully when treating children by ESWL to avoid severe complications. In the data available there is no evidence of long-term side effects on kidney function in children, but the number of study samples is low and should be further studied. Early complications are described more often; this was also the focus of the present paper [9, 10].

Initially, young age was considered a contraindication to ESWL treatment, yet this view changed after Newman’s et al publication in 1980 [11]. Since then, an increase in the number of ESWL procedures among the paediatric population has been observed.

The effectiveness of ESWL treatment is usually determined by the total or almost complete (small fragments remaining) expulsion of the deposit and is assessed several months after the procedure. The analysis by Nazli showed ESWL to be effective in 67.9% to 92.85% of the cases [12]. The Polish population was studied by Bar, where the effectiveness of ESWL ranged from 50 to 95% depending on the type of lithotriptor, and the size and location of the deposits [13]. Long-term follow-up and determining the effectiveness of the ESWL procedure was not the aim of our study. It was, therefore, not assessed.

The focus of the work presented was to analyse the early complications of the procedure. However, those complications (which result from the displacement of the calculi fragments) could only have been assessed among treatments where deposit disintegration was achieved. Hence, only the procedures effective in that way were taken into account – 94.3% (233 out of 247 treatments), which is comparable with the results achieved in other studies. It must be noted that ESWL is considered to be more effective among the paediatric than the adult population. This may probably be explained by a more fragile calculi structure, a shorter, more flexible, elastic ureter and less difficulty in passing disintegrated stone particles. Furthermore, less energy is required to disintegrate the calculi, due to children’s thinner skin and smaller body volume [9, 10].

Urolithiasis in the paediatric population was assumed to be more common among boys (the male to female ratio is 1.2:1) [3, 12]. However, recent research reports no sex predominance, and even suggests higher disease incidence among teenage girls. Our records also showed female prevalence (1.23:1).

Early complications after ESWL are rare; they occur in 3 to 7% of the cases and are usually mild and transient [14]. These include mainly: skin lesions, haematoma, oedema of the kidney parenchyma and are an effect of using shockwaves. Other complications include urinary obstruction caused by deposit fragments and require urological intervention. Clinical complications mainly manifest with haematuria, “steinstrasse” in the radiograph, renal colic or urinary tract infection [9, 10, 15].

In our research complications occurred in 15.02% (35) ESWL procedures, which is comparable to other studies. [14,15]. No skin lesions, haematomas, renal oedema were reported. However, 10.73% (25) procedures resulted in a significant dilatation of the urinary tract, which did not diminish with conservative treatment (antispasmodic drugs and alpha blockers) and required a URSL treatment (in two cases double-J stents were also placed) (Table I). Nevertheless, the benefits and potential of ESWL procedure outweigh the significant number of surgical complications.

**Table I:** Early complications observed in the group studied after ESWL. Tabela I. Wczesne powikłania obserwowane w badanej populacji po zabiegu ESWL.

Complication type *Rodzaj powikłania*	Number of patients (%) *Liczba pacjentów (%)*
Total *Wszystkie* *powikłania*	35 (100%)
Urinary obstruction *Blokada odpływu moczu*	22 (62.8%)
Urinary tract infection *ZUM* Severe abdominal pain *Silny ból brzucha*	7 (20%) 3 (8.6%)
Urinary obstruction + UTI *Blokada odpływu moczu + ZUM*	3 (8.6%)

UTI – urinary tract infection ZUM − zakażenie układu moczowego

No serious early complications: ureteral perforation, acute pancreatitis, rupture of an aortic aneurysm, hepatic or splenic haematoma, gastrointestinal bleeding were observed in the study group. Therefore, it can be assumed that the treatment was performed in a safe manner, which confirms the conclusions of other studies [10, 16].

One limitation of this retrospective study is the short observation period. Moreover, we did not analyse the patient’s age, size of the stones, their location and influence on the number of complications, but we plan to extend our observations in the future.

**Table II:** ESWL procedures performed on patients with potentially worse urine flow. Tabela II. Zabiegi ESWL przeprowadzone u pacjentów z potencjalnie gorszym spływem moczu.

	Number of treatments (%) *Liczba zabiegów (%)*
Total *Wszystkie obciążenia*	17 (100%)
Past of the surgery urethropelvic	8 (47.06 %)
junction Stan po operacji zwężenia *podmiedniczkowego*	
Duplication of the PCS *Zdwojenie UKM*	4 (23.53 %)
Duplication of the PCS and ureter *Zdwojenie i moczowodu UKM*	2 (11.76 %)
Vesicoureterel reflux IV° *OPM IV stopnia*	2 (11.76 %)
Horseshoe kidney *Nerka podkowiast*	1 (5.89 %)

PCS − pelvicalyceal system UKM − układ kielichowo-miedniczkowy OPM − odpływ pęcherzowo-moczowodowy

## Conclusion

The vast majority of complications observed in the study were associated with urinary obstruction caused by a partial blockage of the ureter by disintegrated fragments of kidney stones. Despite the complications documented, ESWL remains a safe procedure on condition that it is possible to perform minimally invasive endoscopic procedure if necessary.
